# Tumor-associated exosomes promote lung cancer metastasis through multiple mechanisms

**DOI:** 10.1186/s12943-021-01411-w

**Published:** 2021-09-13

**Authors:** Chunyang Jiang, Na Zhang, Xiaoli Hu, Hongyan Wang

**Affiliations:** 1 Department of Thoracic Surgery, Tianjin Union Medical Center, Nankai University, 190 Jieyuan Road, Hongqiao District, Tianjin, 300121 China; 2 Department of Respiratory Medicine, Tianjin Union Medical Center, Nankai University, 190 Jieyuan Road, Hongqiao District, Tianjin, 300121 China; 3 Department of Respiratory Medicine, The Second People’s Hospital of Linhai City, 198 Dubei Road, Linhai, 317016 Zhejiang Province China; 4 Department of Thoracic Surgery, The 4th Hospital of Hebei Medical University, 12 Jiankang Road, Shijiazhuang, 050011 Hebei Province China

**Keywords:** Lung cancer, Exosomes, Metastases, Diagnosis, Therapeutic targets

## Abstract

As an important medium of intercellular communication, exosomes play an important role in information transmission between tumor cells and their microenvironment. Tumor metastasis is a serious influencing factor for poor treatment effect and shortened survival. Lung cancer is a major malignant tumor that seriously threatens human health. The study of the underlying mechanisms of exosomes in tumor genesis and development may provide new ideas for early and effective diagnosis and treatment of lung cancer metastasis. Many studies have shown that tumor-derived exosomes promote lung cancer development through a number of processes. By promoting epithelial–mesenchymal transition of tumor cells, they induce angiogenesis, establishment of the pretransfer microenvironment, and immune escape. This understanding enables researchers to better understand the mechanism of lung cancer metastasis and explore new treatments for clinical application. In this article, we systematically review current research progress of tumor-derived exosomes in metastasis of lung cancer. Although positive progress has been made toward understanding the mechanism of exosomes in lung cancer metastasis, systematic basic research and clinical translational research remains lacking and are needed to translate our scientific understanding toward applications in the clinical diagnosis and treatment of lung cancer metastasis in the near future.

## Statement of significance

1. Tumor-derived exosomes promote lung cancer development through a number of processes.
2. In this article, we systematically review the current research progress of tumor-derived exosomes in metastasis of lung cancer.
3. These findings about exosomes in tumor genesis and development may provide new ideas for diagnosis and treatment of lung cancer metastasis.

## Introduction

Extracellular vesicles (EVs) are lipid bilayer-enclosed extracellular structures which can be formed by outward budding of the plasma membrane or by an intracellular endocytic trafficking pathway involving fusion of multivesicular late endocytic compartments with the plasma membrane. These fusion events result in the extracellular release of the intraluminal vesicles of these compartments, generating a subtype of EVs termed ‘exosomes’ [1]. Information transmission between tumor cells and various cells in the microenvironment plays an important role in tumor metastasis, and exosomes are one of the important mediums of intercell communication [1, 2]. Exosomes are vesicles with a diameter of 30–100 nm secreted by different types of cells [3]. They carry many kinds of substances, such as lipids, nucleic acids, and proteins, and are widely distributed in body fluids, including urine, plasma, lavage fluid, serosal effusion, and cerebrospinal fluid [4]. Exosomes have important roles in multiple physiological and pathological processes, exerting biological functions. On the one hand, they are necessary to maintain normal physiological responses. On the other hand, in the pathological state, especially in the tumor environment, they promote carcinogenesis, proliferation, migration, invasion, immunosuppression, and angiogenesis as well as reshape the microenvironment [5].

In recent years, the study of exosomes in tumor has received enormous interest. Exosomes contain bio-macromolecules to participate in information exchange between cells [6]. They can increase the invasion ability of tumor cells and promote tumor metastasis, which has become a research hotspot in the field of cancer recently [7, 8]. Different types of tumor cells secrete different exosome contents. Additionally, factors affecting cell homeostasis, such as a hypoxic microenvironment, survival pressure, and chemotherapy drugs, induces tumor cells to secrete exosomes [9, 10]. Therefore, the volume of exosomes secreted by tumors is much higher than that of normal cells. Although the role of most exosomal compounds in cancer is unclear, previous studies have shown that tumor-derived exosomes promote tumor growth and metastasis by inducing epithelial–mesenchymal transformation (EMT) of tumor cells. They also promote angiogenesis, the transformation of cancer-associated fibroblasts, immunosuppression, and formation of a premetastatic microenvironment by acting on stromal cells in the microenvironment [11–15].

**Fig. 1:**
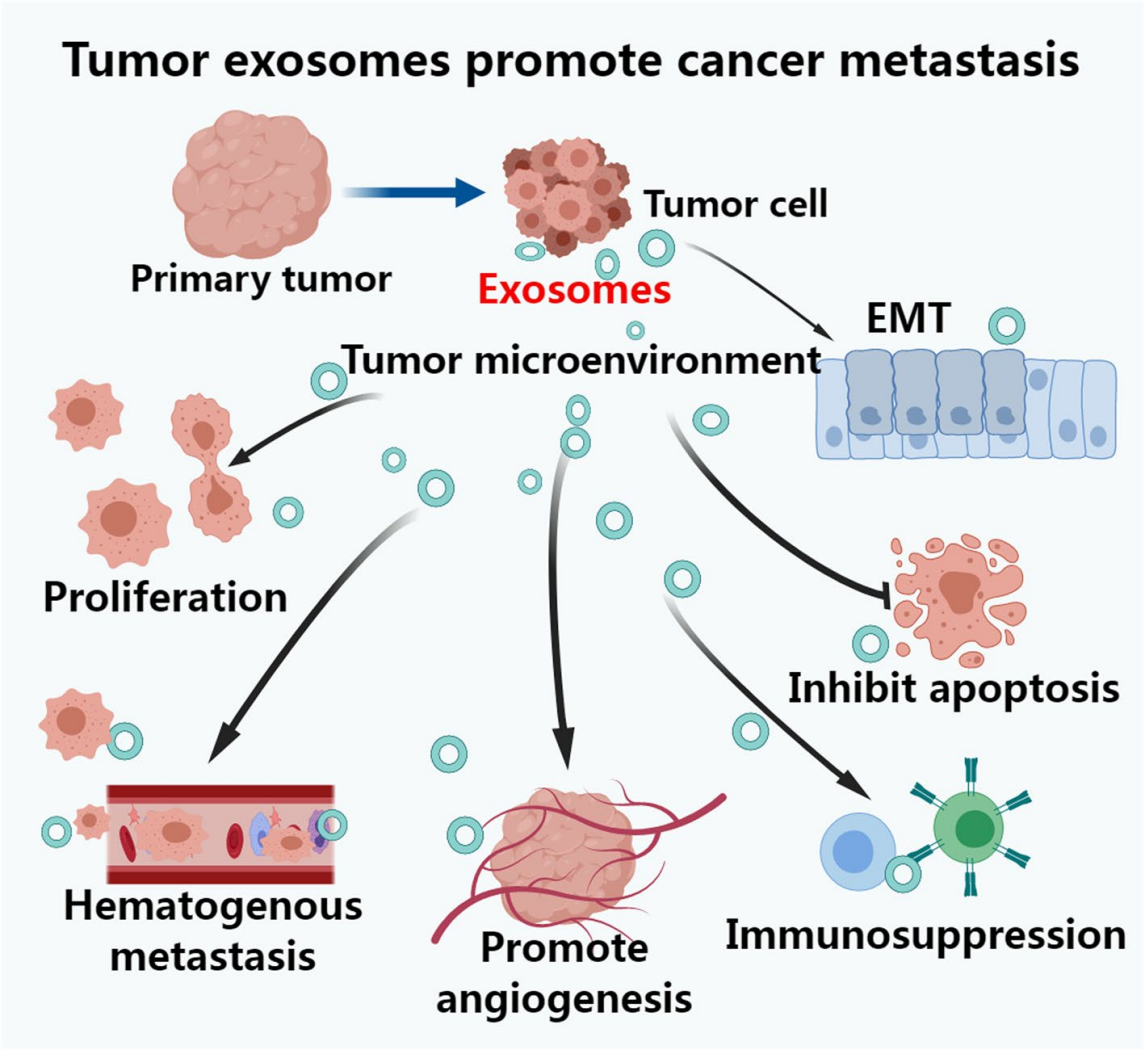
Tumor-derived exosomes promote cancer metastasis. Tumor-derived exosomes through multiple mechanisms participate in cancer metastasis by reshaping the tumor microenvironment; promoting cellular epithelial–mesenchymal transformation (EMT); promoting cell proliferation, inhabiting apoptosis; immunosuppression; promoting hematogenous metastasis and angiogenesis of metastasitic tumor to promote cancer metastasis

Several studies have shown that differential expression of exosome contents is closely related to lung cancer metastasis, playing an important role in the multilink and multistep process [16, 17]. The multiple mechanisms of tumor-derived exosomes promoting cancer metastasis are mainly summarized in Fig. 1. The abscission of cancer cells is essentially a manifestation of increased migration and invasion of tumor cells. Compared with those in healthy people, exosomes are more abundant in circulating body fluids of patients with lung cancer. A number of studies have found that exosomes promote the occurrence and development of lung cancer by promoting the formation of the lung cancer microenvironment, increasing the ability of tumor cell invasion and metastasis, mediating tumor immunosuppression, and participating in chemo-radiotherapy resistance [18]. The study of the underlying mechanisms of exosomes in tumor genesis and development may provide new ideas for early and effective diagnosis and treatment of lung cancer metastasis. Therefore, in this article, the relevant research status of the role of exosomes in lung cancer migration and invasion, immunosuppression and escape, angiogenesis, and other processes is reviewed.

### Tumor exosomes influence lung cancer metastasis by promoting cellular EMT

Many studies have confirmed that lung cancer cell-derived exosomes have an important biological role in distant metastasis of lung cancer by promoting the EMT process in normal epithelial cells. Additionally, mesenchymal stem cells (MSCs), cancer stem cells (CSCs) and other exosome-producing cells can promote the EMT process of cells (summarized in Table 1).

**Table 1:** Exosomes related with lung cancer metastasis by EMT

**Cancer type**	**Exosomes source**	**Related genes or pathway**	**Tissues and/or cells**	**Experimental data**	**Function**	**Refs**
Lung cancer	Cell culture fluid	SNAI1	Lung cancer cells	Promote cells EMT by CAFs deliver SNAI1 to recipient cancer cells via exosomes	Promote EMT	[19]
Lung cancer (NSCLC)	Cell culture fluid	ZEB1	HBEC cell model	Exosomes transfer chemoresistance and mesenchymal phenotypes to recipient cells.	Promote EMT	[20]
Lung cancer (NSCLC)	Cell culture fluid	TRAF4	Lung fibroblasts	By stabilize NOX complex to promote the proliferation and EMT of NSCLC cells	Promote EMT	[21]
Lung cancer	Cell culture fluid	TGF-β1; Smad2/3; Akt/GSK-3β/β-catenin; NF-κB; ERK; JNK and p38 MAPK	MSCs	Promotes EMT, invasion, and migration; enhance the anti-proliferation and pro-apoptotic effect of MSCs on lung cancer cells	Promote EMT	[22]
Lung cancer	Cell culture fluid	TGF-β1; specific miRNAs of exosomes	Lung cancer cells (A549 and H1299 cells)	Promote migration, invasion and expression of mesenchymal markers in the recipient cells	Promote EMT	[23]
Lung cancer	Hypoxic BMSCs fluid	miR-193a-3p; miR-210-3p; miR-5100; STAT3 signaling	Lung cancer cells	Exosomes miRNAs activate STAT3 signaling pathway	Promote EMT	[24]
Lung cancer	Cell culture fluid	miR-210-3p; FGFRL1	Lung CSCs	Promotes EMT, and through miR-210-3p combining FGFRL1 enhance the metastatic ability of lung cancer cells	Promote EMT,	[25]
Lung cancer	Cell culture fluid; Serum		Lung cancer cells and human late stage lung cancer serum	Promote EMT	Promote EMT	[26]

*Abbreviations*: *BMSCs* Bone marrow-derived mesenchymal stem cells, *CAFs* Cancer-associated fibroblasts, *CSCs* Cancer stem cells, *EMT* Epithelial-mesenchymal transition, *FGFRL1* Fibroblast growth factor receptor-like 1, *MSCs* Mesenchymal stem cells, *NSCLC* Non-small cell lung cancer

EMT is an important part of the biological process of tumor metastasis. Cancer-associated fibroblasts (CAFs) contain *SNAI1*, which is delivered to recipient lung cancer cells via exosomes of CAFs to promote EMT. The level of *SNAI1* in exosomes is critical for EMT induction in lung cancer cells [19]. Exosomes from lung cancer mesenchymal cells contain *ZEB1* mRNA, which increases the expression of the EMT major transcription factor *ZEB1* in recipient cells through the transfer of *ZEB1* in exosomes, promoting its transformation from epithelial cells to mesenchymal phenotypes and transferring chemotherapeutic resistance to bronchial epithelial cells [20]. *TRAF4* in normal fibroblasts surrounding non-small cell lung cancer (NSCLC) cells that can promote their proliferation and EMT. *TRAF4* mediates phosphorylated p47-phox to form complexes with NOX2 or NOX4, which increase reactive oxygen species level in vivo and enter the cytoplasm, leading to NF-κB-mediated upregulation of ICAM1 in NSCLC cells [21].

MSCs derived from human umbilical cord can promote EMT and invasion and migration of lung cancer cells, inhibit cell proliferation, and promote cell apoptosis. TGF-β1 expression in MSCs enhances the role of MSCs in promoting EMT and enhances antiproliferative and proapoptotic effects of MSCs on lung cancer cells through MSC-derived exosomes [22].

Furthermore, specific exosomal microRNAs (miRNAs), including miR-193A-3p, miR-210-3p, and miR-5100, are also involved in promoting EMT of epithelial cells by exosomes. The miRNA profile of exosomes may be altered after cellular EMT, and these exosomal miRNAs may in turn promote EMT and the migration and invasion of cancer cells [23]. Therefore, miRNAs specifically expressed in exosomes are associated with EMT and metastasis and may serve as new biomarkers for the EMT process in lung cancer. Bone marrow-derived mesenchymal stem cell (BMSC)-derived exosomal miRNAs, including miR-193A-3p, miR-210-3p, and miR-5100, transferred to epithelial cancer cells under hypoxia promote lung cancer cell invasion and EMT by activating the STAT3 signaling pathway and increasing the expression of mesenchymal-related molecules [24]. Similarly, exosomes derived from lung CSCs promote EMT, migration, and invasion of lung cancer cells by upregulating the expression levels of N-cadherin, Vimentin, MMP-9, and MMP-1, and downregulating the expression of E-cadherin. Exosome-miR-210-3p also promotes lung cancer metastasis by binding FGFRL1 and downregulating its expression [25].

In a study on the effects of exosomes derived from human lung cancer serum, highly metastatic cells, and nonmetastatic cells on recipient human bronchial epithelial cells (HBECs), they found that exosomes from highly metastatic lung cancer cells and serum from patients with advanced lung cancer induce Vimentin expression and EMT in HBECs. The results also confirmed that exosomes from highly metastatic cancer cells and advanced lung cancer serum induce migration, invasion, and proliferation of noncancer receptor cells [26]. Thus, cancer-derived exosomes can be a potential mediator of EMT in recipient cells.

Inhibition of EMT process in recipient cells may be an effective treatment for lung cancer metastasis. Targeting specific genes (such as *SNAI1, ZEB1,* miR-193A-3p, miR-210-3p, etc.) in these tumor-associated exosomes can effectively inhibit the exosome pathway to promote lung cancer metastasis, which may provide a new method for the prevention and treatment of lung cancer metastasis. Based on the important findings of the above studies, how to inhibit EMT specifically and effectively to block lung cancer metastasis as well as how to conduct clinical research and translate it into clinical practice are important questions that will need to be addressed in key future research.

### Tumor exosomes influence lung cancer metastasis by regulating cell proliferation, apoptosis, and migration

Studies have shown that exosomes from lung cancer or other tumor sources play important roles in lung cancer metastasis by promoting the proliferation of lung cancer cells, inhibiting apoptosis, and regulating the invasion and migration ability of lung cancer cells (summarized in Table 2). Wnt3a/β-catenin, *circSATB2*, HIF-1α/COX-2, *KLF9*, and *LMO7* in tumor-derived exosomes regulate genes or signaling pathways to promote proliferation and migration of lung cancer cells. Further, specific miRNAs in tumor-derived exosomes, including miR-326, miR-135b, miR-210, miR-660-5p, and miR-96, play a stimulating role as important regulators in lung cancer cell proliferation and migration [27–31]. Wnt5b overexpression is associated with cancer aggressiveness. Exosomes derived from cells of the human pancreatic cancer cell line PANC-1 activate Wnt5b signaling in Chinese hamster ovary cells and stimulate the migration and proliferation of A549 lung adenocarcinoma (ADC) cells. Caco-2 colon cancer cells were used to establish Caco-2/Wnt5b cells with ectopic expression of Wnt5b, whose exosomes also stimulated the migration and proliferation of A549 cells [32]. These results indicate that *Wnt5b*-associated exosomes promote cell migration and proliferation in a paracrine manner.

**Table 2:** Exosomes related with lung cancer metastasis by regulating cell proliferation, apoptosis and migration

**Cancer type**	**Exosomes source**	**Related genes or pathway**		**Tissues and/or cells**	**Experimental data**	**Functions**	**Refs**
***Promote proliferation and migration***							
Lung cancer	Cell culture fluid	Wnt3a/β-catenin		Lung cancer cells	Exosomes containing high levels of Wnt3a activate β-catenin signaling	Promote proliferation	[27]
Lung cancer (NSCLC)	Serum	circSATB2; miR-326		Lung cancer cells	Regulate FSCN1 expression positively via miR-326 and be transferred by exosomes	Promote proliferation, migration and invasion	[28]
Lung cancer (NSCLC)	Cell culture fluid	HIF-1α/COX-2; miR-135b; miR-210		Lung cancer cells	Hypoxia enhance numbers of exosomes and up-regulate of exosomal HIF-1α/COX-2 and expression of exosomal miR-135b and miR-210	Promote proliferation and migration	[29]
Lung cancer (NSCLC)	Serum	miR-660-5p; KLF9		Lung cancer cells	miR-660-5p in exosome may control NSCLC proliferation, viability, and metastasis by targeting KLF9	Promote proliferation and migration	[30]
Lung cancer	Serum	miR-96; LMO7		Lung cancer patients	Exosomal miR-96 promotes lung cancer progression by targeting LMO7.	Promote proliferation, migration, and drug resistance	[31]
Lung cancer (adenocarcinoma)	Cell culture fluid	Wnt5b		PANC-1 cells	Exosomes activate Wnt5b signaling in CHO cells and stimulate migration and proliferation of lung adenocarcinoma cells A549	Promote proliferation and migration	[32]
***Promote invasion and or migration***							
Lung cancer (Adenocarcinoma)	Lung cancer	TNF-β; TNF-α; IL-6; IL-8; IL-10; CD163; iNOS; MMP2; MMP9		Lung cancer cells	Exosomes derived from lung adenocarcinoma cells can activate macrophages, increase MMP2 and MMP9 levels, and enhance the invasion of lung adenocarcinoma cells.	Promote invasion	[33]
Lung cancer (NSCLC)	Pleura exudates	GGT-1; LTC4; CysLT1		Lung cancer cells	Exosomes contain GGT-1 and transform exogenous LTC4 to pro-tumorigenic LTD4, and elevate the level of endogenous CysLT	Promote survival and migration	[34]
Lung cancer	BALF	E-cadherin		Lung cancer cells	In exosomes from lung cancer, BALF promote the migration and invasion by carrying E-cadherin	Promote migration and invasion	[35]
Lung cancer (SCLC)	Cell culture fluid	FECR1; FECR2; miR584-3p; ROCK1		Lung cancer cells	FECR1 and FECR2 up-regulated in SCLC tissues; exosomal FECRs promotes lung cancer cells metastasis through the miR584-ROCK1 pathway	Promote invasion and migration	[36]
Lung cancer	Serum	miR-106b; PTEN; MMP-2; MMP-9		Lung cancer patients	Exosomal miR-106b target PTEN, increase the MMP-2 and MMP-9 expression and promote lung cancer cell migration and invasion	Promote invasion and migration	[37]
Lung cancer	Cell culture fluid	ALDOA; ALDH3A1		Lung cancer cells	Exosomes carrying ALDOA and ALDH3A1 from irradiated lung cancer cells enhance migration and invasion of recipient cells by accelerating glycolysis	Promote invasion and migration	[38]
Lung cancer	Cell culture fluid	MMP3; MMP9		Adipocytes cells	Adipocyte-derived exosomes promote lung cancer cells metastasis by increasing MMP9 activity via transferring MMP3 to lung cancer cells	Promote invasion and migration	[39]
***Promote proliferation and or inhibit apoptosis***							
Lung cancer (NSCLC)	Cell culture fluid	ASMA		Lung cancer cells; Human lung fibroblasts cell line HLF1 cells	Promote cell proliferation and inhibit cell apoptosis in both normal lung fibroblasts and NSCLC cells by delivering ASMA	Promote proliferation and inhibit apoptosis	[40]
Lung cancer (NSCLC)	Cell culture fluid	MALAT-1		Lung cancer cells	Serum exosome-derived long noncoding RNA MALAT-1 promotes the tumor growth and migration, and prevents tumor cells from apoptosis	Promote proliferation and migration; Inhibit apoptosis	[41]
Lung cancer (SCLC)	HBMECs	S100A16		Lung cancer cells	Elevation of S100A16 prevent the loss of mitochondrial membrane potential and enhance resistance to apoptosis of SCLC cells	Inhibit apoptosis	[42]
Lung cancer	Macrophages cell culture fluid	Let-7a-5p; BCL2L1; PI3Kγ		Macrophages cells	Exogenous let-7a-5p induces lung cancer cell death through BCL2L1-mediated PI3Kγ signaling pathway	Promote autophagic cell death	[43]
***Regulate or inhibit migration***							
Lung cancer	Cell culture fluid	TGF-β; lnc-MMP2-2	Lung cancer cells		TGF-β-mediated exosomal lnc-MMP2-2 might regulate the migration and invasion of lung cancer cells into the vasculature by promoting MMP2 expression	Regulate migration	[44]
Lung cancer	Cell culture fluid	TGF-β; IL-10	Lung cancer cells NCI-H1688		Exosomes derived from cancer cells regulate the cellular migration of tumor cells through TGF-β and IL-10	Regulate migration	[45]
Lung cancer (NSCLC)	Cell culture fluid	PEDF; THBS1	Lung cancer cells		Exosomes from PEDF-treated cells contain THBS1, which inhibit cytoskeletal remodeling and exosome-induced lung cancer cell motility, migration, and invasion	Inhibit invasion and migration	[46]

*Abbreviations*: *ALDH3A1* Aldehyde dehydrogenase 3A1, *ALDOA* Aldolase A, *ASMA* Alpha-smooth muscle actin, *BALF* Bronchoalveolar lavage fluid, *BCL2L1* B-cell lymphoma-2, *CHO* Chinese hamster ovary, *COX-2* Cyclooxygenase-2, *CysLT1* Cysteinyl leukotriene 1, *FECR* FLI1 exonic circular RNAs, *FSCN1* Fascin homolog 1, actin-bundling protein 1, *GGT-1* γ-glutamyl transpeptidase 1, *HBMECs* Human brain microvascular endothelial cells, *HIF-1α* Hypoxia inducible factor-1, *IL* Interleukin, *iNOS* Inducible nitric-oxide synthase, *KLF9* Kriippel-like factor9, *LMO7* LIM-domain only protein 7, *LT* Leukotriene, *MALAT-1* Metastasis-associated lung adenocarcinoma transcript 1, *MMP* Matrix metalloproteinase, *NSCLC* Non-small cell lung cancer, *ROCK1* Rho Associated Coiled-Coil Containing Protein Kinase 1 gene, *PANC-1* Human pancreatic cancer cell line, *PEDF* Pigment epithelium-derived factor, *PI3Kγ* PI-3 kinase gamma, *PTEN* Phosphatase and tensin homolog deleted on chromosome ten, *SCLC* Small cell lung cancer, *TGF* Transforming growth factor, *THBS1* Thrombospondin 1, *TNF* Tumor necrosis factor

mRNA transcripts of *GGT-1*, *LTC4*, *FECR1*, *FECR2*, miR-106b, *ALDOA*, *ALDH3A1*, and other genes in exosomes derived from lung cancer cells act as important regulators by playing an enabling role in promoting the invasion and migration of lung cancer cells. Tumor-derived exosomes derived from lung cancer cells promote the invasion and migration of lung cancer cells by regulating the expression levels of specific genes, including those encoding the matrix metalloproteinases MMP-2, MMP-9, *PTEN*, *E-cadherin*, and *ROCK1* [33–38]. Nontumor exosomes derived from adipocytes increase the activity of MMP9 by delivering MMP3 to lung cancer cells, thereby promoting the invasion and migration of lung cancer cells [39].

Lung cancer cell-derived exosomes also promote lung cancer metastasis by inhibiting apoptosis. These exosomes contribute to lung cancer progression by delivering transcripts of *ASMA*, *S100A16*, and the long noncoding RNA (lncRNA) *MALAT-1*, all of which promote tumor growth and migration and prevent apoptosis [40–42]. In contrast, macrophage cell-derived exosomes contain *let-7a-5p*, which promotes autophagic cell death of lung cancer cells through the *BCL2L1*-mediated *PI3Kγ* signaling pathway [43]. Thus, exosomal *let-7a-5p* plays a significant role in inhibiting tumor invasion and metastasis.

In terms of the simple regulation of lung cancer migration, studies have confirmed that transcripts of *TGF-β*, lnc-MMP2-2, IL-10, as well as those of other genes in exosomes derived from lung cancer cells have regulatory functions. They play a key role in regulating the migration ability of lung cancer cells by targeting and regulating related genes, thus participating in the biological process of lung cancer metastasis [44, 45]. Pigment epithelium-derived factor (PEDF)-treated exosomes from lung cancer cells inhibit cytoskeletal remodeling by secreting *THBS1*, which reduces the vitality of lung cancer cells and inhibits cell invasion and migration [46].

These findings have demonstrated that tumor-derived exosomes participate in the biological process of lung cancer metastasis by regulating cell apoptosis, cell proliferation, and cell migration. It can be predicted that blocking the biological functions of these key genes in tumor-derived exosomes to inhibit apoptosis, promote proliferation and migration will provide a new approach for the treatment of lung cancer metastasis induced by tumor exosomes. However, additional in-depth studies are needed to drive clinical transformation research and new clinical practice in lung cancer metastasis.

### Tumor exosomes influence lung cancer metastasis by regulating immunity and angiogenesis

Many studies have shown that exosomes from lung cancer or other tumor sources can suppress immunity leading to immune escape and promote metastasis of lung cancer cells (Table 3). For instance, exosomes from lung cancer cells express PD-L1 and promote tumor growth by reducing T cell activity to induce immune escape. Exosomal PD-L1 inhibits the secretion of interferon-γ (IFN-γ) by Jurkat T cells. Exosomes impair immune function and promote lung cancer metastasis by reducing cytokine production and inducing apoptosis of CD8^+^ T cells [47]. Exosomes from Lewis lung carcinoma cells block the differentiation of bone marrow progenitor cells into CD11c^+^ dendritic cells (DCs) and induce apoptosis. However, after tumor exosome PD-L1 was blocked, the immunosuppressive ability of DCs was partially restored [48].

**Table 3:** Exosomes related with lung cancer metastasis by the regulation of immune and angiogenesis

**Cancer type**	**Exosomes source**	**Related genes or pathway**	**Tissues and/or cells**	**Experimental data**	**Function**	**Refs**
***Immune regulation***						
Lung cancer (NSCLC)	Cell culture fluid	PD-L1	Lung cancer cells	Reduce T cell activity resulting in immune escape, and promote tumor growth	Immune inhibition; immune escape	[47]
Lung cancer	Lung cancer cells (LLC Lewis)	PD-L1	LLC Lewis lung carcinomacells	Tumor exosome treatment can inhibit the maturation and migration of DCs and promote DCs immunosuppression	Immune inhibition	[48]
Lung cancer	Tumors secreting	miR-21; miR-29a; TLR7; TLR8	Lung cancer cells (A549)	miR-21 and miR-29a bind to TLR7 and TLR8, triggering a TLR-mediated prometastatic inflammatory response that leads to tumor growth and metastasis	Immune inhibition	[49]
Lung cancer	TD-MVs	TGF-β1; miR-23a	Lung cancer cells	TD-MVS transferred TGF-β1 into NK cells and inhibited NK cell function under hypoxia	Immune inhibition	[50]
Lung cancer	Patients serum	EGFR	Lung cancer patients	Induction of immune tolerance in dendritic cells by inhibiting tumor antigen-specific CD8 + T cells	Immune inhibition	[51]
Lung cancer	MDA-MB-231 cell culture fluid	miR-126; PTEN/PI3K/ Akt signaling pathway	Lung cancer cells (A549)	MDA-MB-231 cell-derived exosomes can recognize A549 cells in the blood and effectively escape from in vitro immune monitoring systems; miR-126–231-Exo strongly inhibits the proliferation and migration of A549 cells by blocking the PTEN/PI3K/ Akt signaling pathway	Immune escape	[52]
***Angiogenesis regulation***						
Lung cancer	Cell culture fluid	miR-23a; PHD1; PHD2; ZO-1	Lung cancer cells	miR-23a up-regulated in exosomes of lung cancer under hypoxic conditions. miR-23a inhibits PHD1 and 2, leading to HIF-1 accumulation to enhance angiogenesis and, in addition, inhibits ZO-1, increases vascular permeability and cancer transepithelial migration	Enhance angiogenesis	[53]
Lung cancer	Cell culture fluid (malignant transformation of HBE cells)	exosomal miR-21; STAT3; VEGF	HBE cells	miR-21 in exosomes activates STAT3, increases VEGF levels in recipient cells, and promotes angiogenesis and malignant transformation of HBE cells	Enhance angiogenesis	[54]
Lung cancer	Serum from patients with NSCLC	exosomal miR-126	HBE cells	miR-126 in exosomes induces angiogenesis and malignant transformation of HBE cells.	Enhance angiogenesis	[55]
Lung cancer	Lung cancer cells secreting (Hypoxic conditions)	HIF-1α; COX-2; miR-135b; miR-210	Lung cancer cells (A549)	Hypoxia-induced exosomes can promote the proliferation, migration and angiogenesis of A549 cells. Aspirin attenudes this stimulative effect by inhibiting the proliferation of hypoxic A549 cells, reducing exosome secretion and changing exosome composition.	Enhance angiogenesis	[29]
Lung cancer (SCLC)	NCI-H69 SCLC cells secreting	sFlt-1	HUVECs	Exosomes of SCLC cell lines contain very low level of sFlt-1, which significantly increases the migration of HUVECs and weakens the inhibitory effect of NCI-H69-Exo on angiogenesis.	Enhance angiogenesis	[56]
Lung cancer	TECs (derived from ADC and SCC)	CDH2; MAPK/ERK and MAPK/JNK signaling pathways	Lung cancer patients	CDH2 significantly promoted angiogenesis in vivo and in vitro.	Enhance angiogenesis	[57]

*Abbreviations*: *ADC* Adenocarcinoma, *CDH2* Cadherin-2, *DCs* Dendritic cells, *HBEs* Human bronchial epithelial cells, *HUVEC* Human umbilical vein endothelial cells, *PHD* Prolyl hydroxylase, *TD-MVs* Tumor-derived microvesicles, *TECs* Tumor-derived endothelial cells, *TLR* Toll-like receptor, *NSCLC* Non-small cell lung cancer, *SCC* Squamous cell carcinoma, *SCLC* Small cell lung cancer, *sFlt-1* Soluble fms-like tyrosine kinase-1, *VEGF* Vascular endothelial growth factor

As miRNAs are involved in tumor–immune system communication and play an important role in tumor growth and progression, they are a promising target of cancer therapy. MiR-21 and miR-29a bind to TLR7 and TLR8, thereby stimulating a TLR-mediated prometastatic inflammatory response that leads to tumor growth and metastasis [49]. Tumor-derived microvesicles (TD-MVs) transfer *TGF-β1* to natural killer (NK) cells and decrease the expression of the activated receptor NKG2D on the cell surface, while high levels of miR-210 and miR-23a in hypoxic TD-MVs act as additional immunosuppressors that directly affect the expression of CD107a in NK cells and reduce the antitumor immune response of NK cells [50].

Epidermal growth factor receptor (*EGFR*) is closely related to the occurrence and development of lung cancer. The presence of *EGFR* in lung cancer exosomes induces tolerant DCs. Tolerant DCs and Th0 cells were co-cultured to produce tumor antigen-specific regulatory T cells (Tregs), which inhibit tumor antigen-specific CD8^+^ T cells and cause immune tolerance in patients with lung cancer [51]. Additionally, MDA-MB-231 cell-derived exosomes recognize A549 cells in blood, thereby promoting immune escape and distant metastasis of lung cancer cells [52].

A number of studies have reported that lung cancer exosomes also promote lung cancer cell metastasis by increasing angiogenesis (summarized in Table 3). Lung cancer cell-derived exosomal miR-23a directly inhibits the prolyl hydroxylases PHD1 and PHD2, leading to the accumulation of hypoxia-inducible factor-1 (*HIF-1*) in endothelial cells to enhance angiogenesis. MiR-23a also inhibits the tight junction protein ZO-1, thereby increasing vascular permeability and cancer trans epithelial migration [53]. Further, miR-21 in cigarette smoke extract-transformed HBEC exosomes induces *STAT3* activation, which increases *VEGF* levels in recipient cells and promotes angiogenesis and malignant transformation of HBECs [54]. MiR126 is mainly present in the exosomes of patients with NSCLC. Exosomal miR126 induces angiogenesis and malignant transformation of HBECs [55]. Hypoxia-induced lung cancer stem cell-derived exosomes promote proliferation, migration, and angiogenesis of A549 cells, whereas aspirin attenuates this effect by reducing exosome secretion and altering exosome components (i.e., upregulating HIF-1α/COX-2 and downregulating exosomal miR-135b and miR-210) [29]. Exosomes derived from cells of the SCLC cell line NCI-H69 contain a low level of sFlt-1, which can significantly increase the migration ability of human umbilical vein vascular endothelial cells (HUVECs) and weaken the inhibitory effect of NCI-H69-derived exosomes on angiogenesis. Exosomes rich in sFlt-1 inhibit HUVEC migration induced by NCI-H69-derived exosomes, and thus, may be an effective therapeutic agent for inhibiting SCLC metastasis [56]. Cadherin-2 (CDH2) expression was significantly elevated in tumor-derived endothelial cells derived from both ADC and squamous cell carcinoma (SCC). CDH2 significantly promoted angiogenesis and increased sensitivity to antagonists in vivo and in vitro. The MAPK/ERK and MAPK/JNK signaling pathways may play an important role in CDH2-induced HIF-1α/VEGF-mediated angiogenesis [57].

These studies demonstrate the involvement of tumor-derived exosomes in lung cancer metastasis by regulating immune function and angiogenesis and also provide clues for further research directions toward improving diagnosis and treatment of lung cancer metastasis. By targeting specific genes in tumor-derived exosomes associated with immunosuppression and tumor angiogenesis, blocking or reversing the biological functions of these two aspects, it is expected to have a certain therapeutic effect on the metastasis of lung cancer.

### Other mechanisms

Tumor-derived exosomes also promote tumor progression through other mechanisms, such as via inflammation-related pathways, induction of the EGFR pathway in preosteoclasts, or interactions with adjacent cells. Additionally, exosomes from the serum of patients with lung cancer are closely related to tumor stage and metastatic progression (summarized in Table 4).

**Table 4:** Exosomes related with lung cancer metastasis by other mechanisms

**Cancer type**	**Exosomes source**	**Related genes or pathway**	**Tissues and/or cells**	**Experimental data**	**Function**	**Refs**
Lung cancer	Lung cancer cells	TRIM59; NLRP3;, IL-1β	Lung cancer cells; macrophages	Lung cancer cells-derived exosomal TRIM59 converts macrophages via regulating ABHD5 proteasomal degradation, to activate NLRP3 inflammasome signaling pathway to promote lung cancer progression by IL-1β secretion	Promote lung cancer progression	[58]
Lung cancer (NSCLC)	Lung cancer cells	AREG; EGFR; RANKL	Lung cancer cells	NSCLC-exosomes, containing AREG, induce EGFR pathway activation in pre-osteoclasts that in turn causes an increased expression of RANKL	Promote bone metastasis	[59]
Lung cancer	Cell culture fluid	COX-2	Lung cancer cells; THP-1	COX-2 expression is induced by celecoxib treatment in lung cancer cells and is transferred to neighbor cells via exosomes	Involve in the interaction with neighbor cells	[60]
Lung cancer	Lung cancer cells	lncRNA	Lung cancer cells	Lung cancer exosomes initiate global long non-coding RNA changes in mesenchymal stem cells to inhibit MSCs osteogenic and adipogenic differentiation	Tumor exosomes contribute to interactions between MSCs and tumor cells	[61]
Lung cancer (NSCLC)	Serum	miR-222-3p; SOCS3	Gemcitabine- resistant A549 cells	Exosomic miR-222-3p enhances the proliferation, gemcitabine resistance, migration, invasion, and anti-anoikis of parental sensitive cells by directly targeting the promoter of SOCS3	Enhance proliferation and metastasis	[62]
Lung cancer	Serum	Exosomal miR-21 and miR-155	Nude mouse model with lung cancer	Exosomal miRNAs, miR-21 and miR-155, were significantly upregulated in recurrent tumors compared to primary tumors	Exosomal miRNAs as biomarkers of recurrent lung cancer	[63]
Lung cancer	Plasma; Lung cancer cells		Lung cancer cells and lung cancer patients	Including stage I and II cancer patients, plasma exosomes of 90.7% patients had higher similarity to lung cancer cell exosomes than the average of the healthy controls. Such similarity was proportional to the progression of cancer	Similarity between plasma and lung cancer cell relates to progression of cancer	[64]
Lung cancer (NSCLC)	Plasma		Lung cancer patients	Plasma exosome level correlates with tumor stage and may serve as a prognostic factor for NSCLC	Plasma exosome correlate with tumor stage	[65]
Lung cancer (NSCLC)	Plasma	Exosomal Tim-3 and Galectin-9	Lung cancer patients	High levels of Exo-Tim-3 and Exo-Galectin-9 were all positively correlated with larger tumor size, advanced stages, and more distant metastasis	Correlate with metastasis	[66]
Lung cancer (NSCLC)	Serum	Exo-GAS5	NSCLC patients	Patients with NSCLC with larger tumor size and advanced TNM classification showed lower Exo-GAS5 expression	Exo-GAS5 expression is associated with advanced TNM classification	[67]

*Abbreviations*: *ABHD5* Abhydrolase domain containing 5, *AREG* Amphiregulin, *COX-2* Cyclooxygenase-2, *EGFR* Epidermal growth factor receptor, *GASS* Growth arrest-specific transcript 5, *IL* Interleukin, *MSCs* Mesenchymal stem cells, *NLRP3* NOD-LRR-and pyrin domain-containing protein 3, *NSCLC* Non-small cell lung cancer, *RANKL* Receptor Activator for Nuclear Factor-κ B Ligand, *SERS* Surface-enhanced Raman scattering, *SOCS3* Suppressors-of-cytokine-signaling 3, *THP-1* Human myeloid leukemia mononuclear cells, *TNM* T: extent of the primary tumor; N: lymph node involvement; M: metastatic disease, *TRIM59* Tripartite motif-containing 59

Exosomes derived from lung cancer cells secrete TRIM59 and transform macrophages into tumor-promoting macrophages by regulating ABHD5 proteasome degradation, which activates the NLRP3 inflammasome signaling pathway and promotes the progression of lung cancer by secreting IL-1 [58]. NSCLC-derived exosomes containing amphiregulin (AREG) induce activation of the EGFR pathway in preosteoclast cells, which in turn leads to increased RANKL expression and induced expression of proteolytic enzymes that are known as markers of osteoclast formation, triggering a vicious cycle of osteolytic bone metastasis [59].

Lung cancer cells communicate with neighboring cells via exosomes. For instance, exosomes of lung cancer cells contain COX-2. Celecoxib induces COX-2 expression in lung cancer cells, and high expression of COX-2 in exosomes can be transferred to other cells, thereby participating in interactions with neighboring cells [60]. Previous studies have shown that exosomes derived from lung cancer cells inhibit osteogenic and adipogenic differentiation of MSCs, and lncRNAs in exosomes are involved in the regulation of MSC characteristics [61]. These findings bring new insights into the mechanisms of interaction between tumor cell exosomes and environmental components of MSCs. Exosomes from gemcitabine-resistant cells of the NSCLC cell line A549 contain miR-222-3p, which targets *SOCS3* as a major regulator of gemcitabine resistance and malignancy characteristics. Exosomal miR-222-3p promotes tumor progression by directly targeting the *SOCS3* promoter to enhance proliferation, gemcitabine resistance, migration, and invasion of parental sensitive cells [62]. Thus, exosomal miR-222-3p in serum may be a potential prognostic biomarker for gemcitabine sensitivity in patients with NSCLC.

In a related in vivo study using lung cancer H1299 cells, it was found that levels of miR-21 and miR-155 were significantly higher in relapsed tumors than in primary tumors of nude mouse models of subcutaneous xenotransplantation of primary and recurrent lung cancers. In fact, exosomal miRNA signatures may reflect the true pathological features of lung cancer [63]. Therefore, exosomal miRNAs may be used as biomarkers for noninvasive diagnosis of this disease. In a study of plasma-derived exosomes of 43 patients with stage I and II lung cancer evaluated by surface-enhanced Raman spectroscopy and exploration of the characteristics of exosomes of normal and lung cancer cell lines using deep learning, it was found that 90.7% of patient plasma-derived exosomes had higher similarity with those of lung cancer cells. Furthermore, this similarity was in direct proportion to cancer progression [64]. These results show the considerable potential of combining exosome analysis with deep learning as a liquid biopsy method for early and advanced lung cancer diagnosis and monitoring.

In an association study to determine whether there was a relationship between plasma exosome level and clinical characteristics and prognosis in 208 patients with NSCLC, it was found that exosome levels were significantly associated with tumor stage. Cox proportional risk analysis showed that higher exosome levels were independently associated with poorer overall survival [65]. High levels of exosomal Tim-3 and galectin 9 (also known as LGALS9) in plasma exosomes from patients with lung cancer were positively correlated with tumor size, advanced stage, lymph node metastasis, and distant metastasis. Further, plasma levels of exosomal Tim-3 and LGALS9 are higher in patients with lung SCC than in patients with lung ADC [66]. Additionally, exosomal GAS5 expression was downregulated in the serum of patients with NSCLC, and those patients with large tumors and advanced TNM stage showed lower levels of exosomal GAS5 expression [67]. Thus, exosomal Tim-3, LGALS9, and GAS5 may be ideal noninvasive serum markers to identify patients with early and advanced lung cancer, or even therapeutic targets.

## Discussion

As an important information carrier between cells, exosomes participate in many physiologically important processes of the body. However, they also have a role in pathological conditions. In addition to physiological functions such as cell communication and signal transmission, exosomes also play a role in the regulation of local microenvironment by tumor cells. Tumor metastasis is a complex multi-step process involving cancer cell invasion, vascular survival, attachment and host organ colonization. Study confirmed that tumor-derived exosomes promote tumor metastasis by promoting EMT of tumor cells, inducing angiogenesis, the establishment of a premetastatic microenvironment, immune escape, the formation of a metastatic niche, and organ propensity to guide tumor metastasis [68]. Exosomes affect every step of the tumor metastasis cascade and can be targeted by tumor therapy. Thus, exosomes provide a new direction for researchers to study the mechanism of tumor metastasis and clinical translational research.

Tumor-derived exosomes is the main mechanism of intracellular communication between tumor cells and host cells, enabling tumor cells to adapt to their surroundings to favor an optimal microenvironment for tumor initiation and progression. And tumor-derived exosomes contains a large number of different immune-activating and immunosuppressive factors to support the cellular programming of host cells. Tumor-derived exosomes can also drive metastasis by creating a pre-metastatic niche and directing tumor cells to future metastatic sites [69]. Although exosomes have an important role in the occurrence and development of lung cancer and much exploratory research has been performed on exosomes as an important part of the tumor microenvironment in the context of early diagnosis and treatment of lung cancer, the specific mechanisms of exosomes in tumor evolution remain unclear [70]. There are still many questions of the mechanisms about tumor-derived exosomes and cancer metastasis need to be clarified. Further, the sensitivity and specificity of exosomes in the diagnosis and treatment of lung cancer remain to be improved. Therefore, greater attention to the mechanisms underlying exosomes in tumor progression and efforts in translational medicine research will provide a richer basis and support for a clinical role of exosomes in the early diagnosis and treatment of lung cancer [71].

**Fig. 2:**
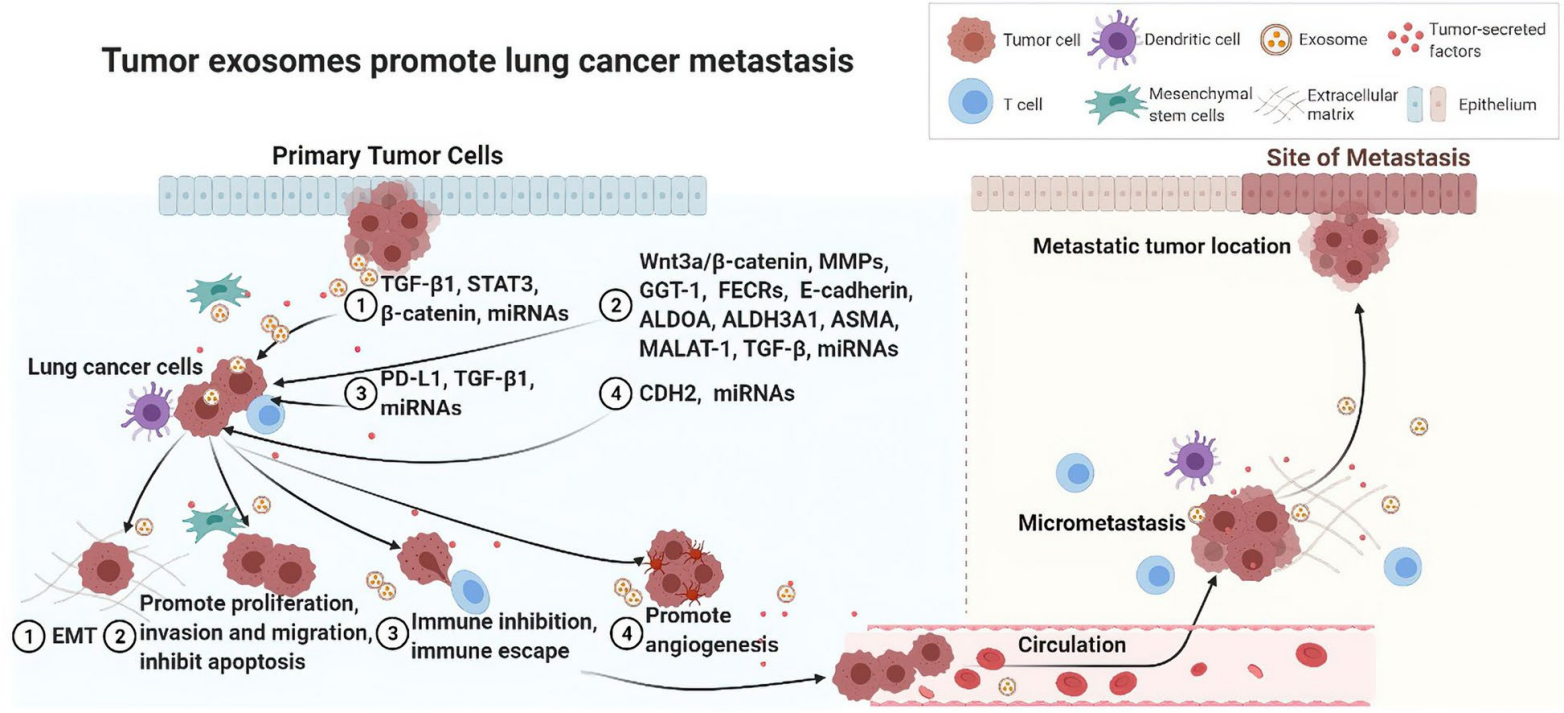
Tumor-derived exosomes promote lung cancer metastasis. Tumor-derived exosomes, especially lung cancer exosomes participate in lung cancer metastasis by promoting cellular epithelial–mesenchymal transformation (EMT); by regulating cell proliferation, apoptosis, and migration; by regulating immune function and angiogenesis; by activating inflammation-related pathways or inducing tumor metastasis signaling pathways; and by interacting with neighboring cells to promote lung cancer progression

This review provides a systematic summary of the current understanding of tumor-derived exosomes, especially lung cancer exosomes in lung cancer metastasis. The studies in this article describe the influence of lung cancer-associated exosomes that range from promoting cellular EMT and exerting an effect on lung cancer metastasis through regulating cell proliferation, apoptosis, and migration to regulating immune function and angiogenesis; activating inflammation-related pathways or inducing tumor metastasis signaling pathways; and interactions with neighboring cells to promote tumor progression (mainly summarized in Fig. 2). These studies are mainly the results of in vitro studies on the interactions between cells through exosomes, but they also include a small number of in vivo studies using animal models and analyses of clinical cases. Importantly, these studies have identified a number of genes of high research significance toward exosomes and lung cancer development, such as *ZEB1*, *TGF-β1*, *KLF9*, *LMO7*, *ASMA*, *S100A16*, *MALAT-1*, *PD-L1*, *EGFR*, *sFlt-1*, *Tim-3*, and *LGALS9*. Additionally, other important genes that are regulated by exosomes and participate in invasion and metastasis of lung cancer were identified, including those encoding MMPs such as MMP-1 and MMP-9, N-cadherin, and Vimentin.

Among the transcripts of regulatory genes contained in these exosomes, exosomal miRNAs are key regulatory factors of gene expression that participate in cell–cell communication in the tumor microenvironment, mediate immune escape, regulate drug resistance, and promote tumor cell metastasis and angiogenesis, thereby affecting the occurrence and development of lung cancer [72]. Targeting tumor-specific exosomal miRNAs provides a new strategy for the clinical treatment of lung cancer metastases [73]. Additionally, as potential noninvasive biomarkers, exosomal miRNAs would be of clinical value in the early diagnosis of lung cancer metastasis [74]. In fact, the involvement of exosomal miRNAs in the occurrence and progression of tumors brings new opportunities for clinical diagnosis and treatment of lung cancer [75]. However, because of the early stage of research in this field, the clinical application of exosomal miRNAs in lung cancer is still in the preliminary stage of exploration. The exosomal miRNAs described in this review as key regulatory genes for lung cancer metastasis include miR-193a-3p, miR-210-3p, miR-5100, miR-326, miR-135b, miR-210, miR-660-5p, miR-96, miR-23a, miR-21, miR126, and miR-135b. Despite the potential of exosomal miRNAs as diagnostic markers of lung cancer metastasis and potential new targets for treatment, because of their wide variety, the potential regulatory mechanisms in the occurrence and development of lung cancer warrant further research.

Exosomes are enriched in circulating body fluids. The continuous development of liquid biopsy technology accelerates the clinical application of exosomes, and exosome contents are expected to become the next generation of emerging biomarkers [76]. Although research on lung cancer exosomes has made a breakthrough, controversies and challenges remain. For instance, exosomes can inhibit tumor metastasis in lung cancer [77]. Thus, they may play a contradictory dual role that needs to be better understood by further studies. Additionally, exosomes are involved in multiple steps and links in the process of lung cancer metastasis, but the most critical and core links need to be determined. Further extensive systematic and in-depth studies are also needed to determine whether differential expression of exosome contents can be monitored and followed up, leading to effective clinical intervention before tumor metastasis occurs.

## Conclusions

Collectively, exosomes exist as a key pathway of information transmission in tumor cells. At present, positive results have been achieved in studies toward understanding the mechanisms of exosomes in lung cancer metastasis; however, in-depth and systematic studies are still lacking. There are still many questions to be clarified regarding the mechanism of tumor-derived exosomes and lung cancer metastasis. For example, how tumor-derived exosomes and lung cancer cells or host cells recognize each other and how vesicle contents are selected to play a role in regulating tumor metastasis. Which is more influential in metastatic tumors than gene signals from exosomes or gene signals expressed by metastatic tumor cells, etc. Additional exploration is needed to broaden the existing limited information known regarding exosomes and lung, and continued efforts in basic and clinical translational research on exosomes and lung cancer metastasis should be intensified. These future studies will provide stronger guidance for the diagnosis and treatment of lung cancer in the future, especially the diagnosis and clinical treatment of lung cancer metastatic progression, and ensure more positive breakthroughs.

## Data Availability

Not applicable.
